# Phase I and pharmacokinetic study of DACA (XR5000): a novel inhibitor of topoisomerase I and II

**DOI:** 10.1038/sj.bjc.6690598

**Published:** 1999-08

**Authors:** C J Twelves, C Gardner, A Flavin, J Sludden, I Dennis, J de Bono, P Beale, P Vasey, C Hutchison, M A Macham, A Rodriguez, I Judson, N M Bleehen

**Affiliations:** CRC Department of Medical Oncology, Alexander Stone Building, Glasgow, Bearsden, G61 1BD, UK; Data Centre, Cancer Research Campaign, 10 Cambridge Terrace, London, NW1 4JL, UK; MRC Clinical Oncology and Radiotherapy Unit, Addenbrooke's Hospital, Cambridge, CB2 2QQ, UK; Royal Marsden Hospital, Institute of Cancer Research, Drug Development Section, Block E, 15 Cotswold Road, Sutton, Surrey, SM2 5NG, UK

**Keywords:** phase I, cytotoxic, pharmacokinetics, topoisomerase I, topoisomerase II

## Abstract

DACA, also known as XR5000, is an acridine derivative active against both topoisomerase I and II. In this phase I study, DACA was given as a 3-h intravenous infusion on 3 successive days, repeated every 3 weeks. A total of 41 patients were treated at 11 dose levels between 9 mg m^−2^ d^−1^ and the maximum tolerated dose of 800 mg m^−2^ day^−1^ . The commonest, and dose-limiting, toxicity was pain in the infusion arm. One patient given DACA through a central venous catheter experienced chest pain with transient electrocardiogram changes, but no evidence of myocardial infarction. At the highest dose levels, several patients also experienced flushing, pain and paraesthesia around the mouth, eyes and nose and a feeling of agitation. Other side-effects, such as nausea and vomiting, myelosuppression, stomatitis and alopecia, were uncommon. There was one minor response but no objective responses. DACA pharmacokinetics were linear and did not differ between days 1 and 3. The pattern of toxicity seen with DACA is unusual and appears related to the mode of delivery. It is possible that higher doses of DACA could be administered using a different schedule of administration. © 1999 Cancer Research Campaign


*British Journal of Cancer* (1999) **80**(11), 1786–1791
© 1999 Cancer Research Campaign
Article no. bjoc.1999.0598

### Phase I and pharmacokinetic study of DACA (XR5000): a novel inhibitor of topoisomerase I and II

**CJ Twelves[1], C Gardner[2], A Flavin[3], J Sludden[1], I Dennis[3], J de Bono[1], P Beale[4], P Vasey, C Hutchison[1], MA Macham[1], A Rodriguez[4], I Judson[4] and NM Bleehen[3] for the CRC Phase I/II Committee**

[1]CRC Department of Medical Oncology, Alexander Stone Building, Garscube Estate, Bearsden, Glasgow G61 1BD, UK; [2]Data Centre, Cancer Research Campaign, 10 Cambridge Terrace, London NW1 4JL, UK; [3]MRC Clinical Oncology and Radiotherapy Unit, Addenbrooke's Hospital, Cambridge CB2 2QQ, UK; [4]Royal Marsden Hospital, Institute of Cancer Research, Drug Development Section, Block E, 15 Cotswold Road, Belmont, Sutton, Surrey SM2 5NG, UK

**Summary** DACA, also known as XR5000, is an acridine derivative active against both topoisomerase I and II. In this phase I study, DACA was given as a 3-h intravenous infusion on 3 successive days, repeated every 3 weeks. A total of 41 patients were treated at 11 dose levels between 9 mg $m^{-2}$ $d^{-1}$ and the maximum tolerated dose of 800 mg $m^{-2}$ $day^{-1}$ . The commonest, and dose-limiting, toxicity was pain in the infusion arm. One patient given DACA through a central venous catheter experienced chest pain with transient electrocardiogram changes, but no evidence of myocardial infarction. At the highest dose levels, several patients also experienced flushing, pain and paraesthesia around the mouth, eyes and nose and a feeling of agitation. Other side-effects, such as nausea and vomiting, myelosuppression, stomatitis and alopecia, were uncommon. There was one minor response but no objective responses. DACA pharmacokinetics were linear and did not differ between days 1 and 3. The pattern of toxicity seen with DACA is unusual and appears related to the mode of delivery. It is possible that higher doses of DACA could be administered using a different schedule of administration.

**Keywords:** phase I; cytotoxic; pharmacokinetics; topoisomerase I; topoisomerase II

The DNA topoisomerase enzymes I and II are recognized as important targets for cytotoxic drugs. The anthracyclines and epidophyllotoxins are widely used topoisomerase II inhibitors whereas the camptothecins have been developed more recently as pure inhibitors of topoisomerase I. Unlike other agents in clinical use, DACA (N-[2-(dimethylamino)ethyl] acridine-4-carboxamide dihydrochloride), also known as XR5000, is a novel inhibitor of both topoisomerase I and II.

DACA is one of a series of tri-cyclic carboxamide cytotoxic agents that have been developed by the Cancer Research Laboratory in Auckland, New Zealand (Atwell et al, 1987). In addition to interacting with both topoisomerase I and topoisomerase II, DACA has a number of features indicating that it may be clinically important. First, DACA is able to circumvent several types of drug resistance. Secondly, DACA is highly active against certain solid tumour models in vitro and in vivo. Thirdly, DACA appears to cross the blood–brain barrier in animals. Finally, DACA has linear pharmacokinetics in preclinical models, is water soluble, easily formulated and chemically stable.

The structure of DACA is shown in Figure 1. Its formula is $C_{18}H_{19}N_3O.2HCl$ and molecular weight is 366.29. DACA binds to DNA by intercalation and stimulates the formation of cleavable complexes between DNA and topoisomerase I and II. It is distinguishable from amsacrine, another acridine derivative, by its action on topoisomerase I and by the DNA sequence selectivity of cleavage in the presence of topoisomerase II (Schneider et al, 1988).

The activity of DACA against both topoisomerase I and II also distinguishes it from other topoisomerase inhibitors in clinical use. Moreover, DACA maintains activity against a multi-drug resistant subline of the P388 leukaemia expressing the P-glycoprotein (Baguley et al, 1990). Similarly, sublines of the Jurkat human leukaemia cell line, which have reduced amounts of topoisomerase II, are highly resistant to etoposide and doxorubicin, but retain sensitivity to DACA (Finlay et al, 1993). The pattern of activity of DACA in vitro against a variety of human and mouse cell lines, including primary human melanoma cultures derived from fresh surgical melanoma specimens, is distinct from that of amsacrine and etoposide (Marshall et al, 1992; Finlay et al, 1993) with $IC_{50}$ values between 0.09 μM and 3.4 μM (Finlay et al, 1993).

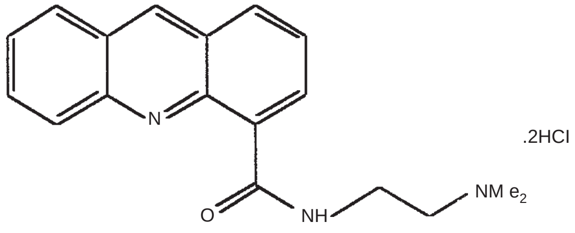

**Figure 1** Structure of DACA

*Received 30 July 1998*
*Revised 19 January 1999*
*Accepted 20 February 1999*

*Correspondence to:* CJ Twelves

DACA has only moderate activity in vivo against P388 leukaemia and L1210 leukaemia. It is, however, highly active against Lewis lung carcinoma in mice (Atwell et al, 1987; Finlay and Baguley, 1989), the murine 16/C tumour and Colon 11 A and Colon 38 tumours (Baguley et al, 1995). In preclinical pharmacology studies the unbound fraction of DACA in mouse plasma was 15.8%. DACA was extensively metabolized and demonstrated biphasic elimination, predominantly in the faeces (Evans et al, 1992; Paxton et al, 1992, 1993*a*, 1993*b*). Tissue levels of DACA were substantially higher in brain, liver, kidney and heart, than in plasma.

The principle objective of this study was to determine the maximum tolerated dose (MTD) of DACA given intravenously (i.v.) over 3 successive days repeated every 3 weeks in patients with solid tumours. The other aims were to determine the safety profile of DACA, study the pharmacokinetics of DACA and describe any evidence of anti-tumour activity.

### MATERIALS AND METHODS

This study was conducted under the auspices of the Cancer Research Campaign Phase I/II Clinical Trials Committee at Addenbrooke's Hospital (Cambridge), the Beatson Oncology Centre (Glasgow) and the Royal Marsden Hospital (Sutton). The study protocol and informed consent forms were reviewed and approved by the Ethics Committee at each hospital; written informed consent was obtained from each patient prior to study entry.

### Study design

Doses of DACA were calculated as mg salt per $m^2$ of body surface area. The starting dose was based on murine data. The $LD_{10}$ dose in mice for a single i.v. dose was 58 mg/kg and for five consecutive daily i.v. doses was approximately 30 mg $kg^{-1}$ per day. The starting dose was 9 mg $m^{-2}$ given daily on 3 successive days, 1/10 of the mouse $LD_{10}$. Dose escalation was according to a modified Fibonacci scheme.

Two patients were entered at the first dose level and at least three patients at further dose levels. If one of these patients experienced a dose-limiting toxicity (DLT), the number of patients treated at that dose level was increased to six. There was an interval of at least 1 week between the entry of patients at any one dose level. Entry of patients at the next dose level was delayed 3 weeks in order that toxicity could be assessed prior to escalation.

The MTD was defined as the dose at which DLTs occur in 33% or more of patients. DLTs were defined as (i) white blood count nadir of less than $0.5 \times 10^9$ $l^{-1}$ or a platelet nadir less than $50 \times 10^9$ $l^{-1}$, (ii) failure to recover by day 35 after the previous treatment to a granulocyte count of $1.5 \times 10^9$ $l^{-1}$ or platelet count of $100 \times 10^9$ $l^{-1}$, (iii) common toxicity criteria (CTC) grade 3 or grade 4 non-haematological toxicity (except for alopecia or nausea and vomiting) unless considered unequivocally to be unrelated to DACA.

### Patient population

Prior to study entry all patients had a baseline history and full physical examination with electrocardiogram (ECG), radiological and laboratory evaluation.

Inclusion criteria included:

i. histologically confirmed metastatic solid tumour refractory to conventional therapy, or for which there was no standard therapy
ii. age ≥ 18 years
iii. WHO performance status 0, 1 or 2 and life expectancy of at least 3 months
iv. adequate haemopoetic reserve, defined as a granulocyte count $\geq 2 \times 10^9$ $l^{-1}$ and platelet count $\geq 100 \times 10^9$ $l^{-1}$
v. adequate renal function, defined as serum creatinine ≤ 0.14 mmol $l^{-1}$
vi. adequate hepatic function, defined as serum bilirubin ≤ 1.5 × the upper limit of normal, with transaminases and alkaline phosphatase ≤ 2 × upper limit of normal and normal prothrombin time.

Major exclusion criteria were:

i. pregnancy, lactating or not taking adequate contraceptive precautions,
ii. chemotherapy or radiotherapy within the previous 4 weeks (at least 6 weeks since treatment with nitrosureas or mitomycin C)
iii. major surgery within the previous 14 days; intercurrent or serious infection within the previous 28 days
iv. life-threatening illness unrelated to cancer.

Whilst on treatment, patients underwent weekly clinical review, performance status was recorded and blood taken for full blood count and biochemistry. Other investigations including ECG and chest X-ray were repeated if clinically indicated. Treatment toxicity was assessed using the National Cancer Institute Common Toxicity Criteria and response evaluated according to WHO criteria.

### Treatment

The concentration of DACA, based on the dihydrochloride salt, was 44 mg $ml^{-1}$. However, all DACA doses are based on the nominal concentration of 50 mg $ml^{-1}$, which represents the concentration of the raw material containing 12% water.

DACA was supplied by the Cancer Research Laboratory, Auckland Medical School, Auckland, New Zealand and formulated at the CRC Formulation Unit at Strathclyde University. The diluted drug is stable at room temperature for at least 6 months. DACA was provided to the clinical centres as an aqueous solution, adjusted to pH 7.1 with sodium hydroxide, in ampoules containing 1.1 ml, 2.2 ml, 5.5 ml at a nominal concentration of 50 mg $ml^{-1}$. DACA was further diluted and administered in 500-ml isotonic saline unless otherwise stated.

The infusion was to run over 3 h, using an infusion pump with i.v. access via a plastic peripheral venous cannula, on 3 successive days. Treatment was to be repeated every 21 days, provided the granulocyte count was $\geq 1.5 \times 10^9$ $l^{-1}$ and platelets $\geq 100 \times 10^9$ $l^{-1}$. Patients who experienced unacceptable toxicity, had progressive disease, or withdrew their consent, did not receive further DACA.

### Pharmacokinetics

Blood samples of approximately 7 ml were taken from an indwelling venous cannula into a heparinized tube and centrifuged at 2000 rpm for 5 min. The plasma was separated and frozen at

© 1999 Cancer Research Campaign

**Table 1** Patient characteristics

| | |
|---|---|
| No. of patients | 41 |
| Male/female | 27/14 |
| Median age (range) | 55 years (31–73) |
| Performance status | |
| 0 | 8 |
| 1 | 26 |
| 2 | 7 |
| Prior non-surgical treatment | |
| None | 1 |
| Chemotherapy | 19[a] |
| Radiotherapy | 11[b] |
| Radiotherapy & chemotherapy | 8* |
| Endocrine or immunotherapy | 2 |
| Tumour type | |
| Lung carcinoma | 13 |
| Colorectal carcinoma | 7 |
| Soft tissue sarcoma | 6 |
| Renal carcinoma | 5 |
| Ovarian carcinoma | 4 |
| Other | 6 |

[a]Two also had endocrine/immunotherapy; [b]Three also had immunotherapy.

–20°C pending assay. On day 1 of the first infusion a total of 18 samples were taken at the following times: 0 (before infusion), 30 min, 1 h, 1 h 30 min, 2 h, 2 h 30 min, 3 h (end of infusion), 3 h 10 min, 3 h 20 min, 3 h 45 min, 4 h, 4 h 30 min, 5 h, 6 h, 8 h, 12 h, 18 h and 24 h. On day 3 this schedule was repeated with additional samples at 48 h, 72 h, 96 h and 120 h. Where the infusion time was prolonged beyond 3 h, an end of infusion sample was collected.

DACA concentrations were determined by high-performance liquid chromatography (HPLC). In brief, 200 µl of plasma were added to 1 ml of acetonitrile and internal standard, mixed and centrifuged at 10 000 rpm for 10 min at 4°C. The supernatant was dried, the residue suspended in 200 µl of mobile phase and centrifuged for 3 min at 10 000 rpm then the supernatant injected onto the HPLC. The system used a Brownlee RP18 precolumn and Phenomenex Primasphere 5 µm C18, 250 mm analytical column. The mobile phase was 28% acetonitrile and 10 mM TEAP (aqueous triethylamine solution adjusted to pH 2 with orthophosphoric acid; 1 M stock diluted 1:100). The flow rate was 1 ml per min and fluorescence detection was used (excitation 360 nm, emission > 450 nm). The assay was linear between 0.01 µM and 10 µM and the coefficient of variation was < 10% for all quality control samples. All pharmacokinetic data are expressed in terms of the dihydrochloride salt.

Both compartmental and non-compartmental pharmacokinetic parameters were calculated using WinNonLin (Scientific Consulting Inc., North Carolina, USA). The area under the concentration-time curve $(AUC)_{0 \to \infty}$ of DACA was calculated using the linear trapezoidal rule with extrapolation to infinity. Comparable $AUC_{0 \to \infty}$ from both compartmental and model independent (non-compartmental) analysis indicated that the choice of model was correct. The selection of best fit was made by comparing residual plots, the Akaike Information Criteria and the standard errors (s.e.) of the parameter estimates.

The data were initially modelled with non-compartmental analysis to give the following parameters: $AUC_{0 \to \infty}$, mean retention time (MRT), clearance (Cl), elimination half-life ($T_{1/2ß}$), peak concentration ($C_{max}$), time to reach peak concentration ($T_{max}$) and volume of distribution at steady state ($V_{ss}$). The data were subsequently modelled using both one-compartment and two-compartment models with uniform weighting.

### RESULTS

### Patients

Forty-one patients were treated in cohorts of between 3 and 9 at 11 dose levels between 9 mg m$^{-2}$ and 800 mg m$^{-2}$. Their clinical characteristics are shown in Table 1. Patients were typical of those included in phase I studies. The commonest tumour types were lung carcinoma, colorectal carcinoma, renal carcinoma and soft tissue sarcoma. Most patients had received at least one non-surgical treatment prior to study entry. The 41 patients received a total of 70 courses of DACA (median 2, range 1–6), although 5 did not receive a full course of three infusions.

### Maximum tolerated dose

The MTD for DACA given as a 3 h i.v. infusion was 800 mg m$^{-2}$ d$^{-1}$. At this dose level all patients experienced significant toxicity. The DLT was pain during the infusion of DACA. This cohort was expanded to nine patients in order to define better the pattern of toxicity. In all, at the 800 mg m$^{-2}$ dose level four patients stopped DACA because of toxicity and a further two experienced unacceptable toxicity. Of the nine patients treated at 800 mg m$^{-2}$, one developed severe chest pain (see below), four had moderate and three had severe arm pain. Prophylactic administration of dexamethasone, nifedipine or analgesics did not prevent the pain. It still occurred when the infusion volume was increased from 500 ml to 1 litre. Five patients also developed a rash or flushing over the upper trunk or face. Six patients reported oral/perioral paraesthesiae or burning, whilst three experienced either lacrimation or pressure behind the eyes.

Also at the 800 mg m$^{-2}$ dose level, one patient developed thrombosis of the right innominate vein confirmed by upper limb venography. She had, however, a past history of left innominate vein thrombosis with residual stenosis that may have predisposed her to further thrombosis. Grade 3 diarrhoea and grade 3 vomiting were each experienced by one patient.

### Toxicities

Important toxicities considered almost certainly, probably or possibly related to treatment in each patient at that dose level are shown in Table 2. There were two deaths following the first cycle of DACA. A 73-year-old man with metastatic melanoma died at home 15 days after treatment with DACA 36 mg m$^{-2}$ d$^{-1}$; post-mortem examination showed left ventricular hypertrophy. The second, a 72-year-old male with lung cancer, was not myelosuppressed but developed a chest infection and died 10 days after treatment with 250 mg m$^{-2}$ d$^{-1}$ DACA. Neither was attributed to DACA by the investigator.

At dose levels up to and including 600 mg m$^{-2}$ d$^{-1}$ DACA was generally well-tolerated although pain at or proximal to the infusion site was seen at all doses. There was, however, considerable variability in the severity of the arm pain between patients at each dose level. There were also differences between infusions in the same patient. Some patients described ‘burning’ at the site of the

 © 1999 Cancer Research Campaign

**Table 2** Worst toxicity experienced by each patient according to dose level

| Dose level (mg m$^{-2}$ day$^{-1}$) | No. of patients | Nausea | Vomiting | Diarrhoea | Stomatitis | Neuro-cortical | Skin | Pain-infusion arm | Agitation | Paraesthesia of head/neck/mouth |
|---|---|---|---|---|---|---|---|---|---|---|
| | | 0 1 2 3 4 | 0 1 2 3 4 | 0 1 2 3 4 | 0 1 2 3 4 | 0 1 2 3 4 | 0 1 2 3 4 | 0 1 2 3 4 | 0 1 2 3 4 | 0 1 2 3 4 |
| 9 | 2 | 2 | 2 | 2 | 2 | 2 | 2 | 1 1 | 2 | 2 |
| 18 | 3 | 2 1 | 3 | 3 | 3 | 3 | 3 | 2 1 | 3 | 3 |
| 36 | 3 | 1 2 | 3 | 3 | 3 | 2 1 | 3 | 1 1 1 | 3 | 3 |
| 60 | 4 | 3 1 | 4 | 3 1 | 4 | 4 | 4 | 1 1 2 | 4 | 4 |
| 100 | 4 | 4 | 4 | 4 | 4 | 4 | 4 | 1 1 2 | 4 | 4 |
| 165 | 3 | 3 | 3 | 3 | 3 | 3 | 3 | 1 2 | 3 | 3 |
| 250 | 3 | 3 | 3 | 3 | 3 | 3 | 3 | 1 2 | 3 | 3 |
| 350 | 3 | 1 1 1 | 3 | 3 | 3 | 3 | 3 | 1 2 | 3 | 3 |
| 450 | 3 | 3 | 3 | 3 | 3 | 3 | 3 | 3 | 3 | 3 |
| 600 | 5 | 2 1 2 | 3 1 1 | 4 1 | 4 1 | 5 | 4 1 | 2 3 | 5 | 3 1 1 |
| 800 | 9 | 6 3 | 6 3 | 7 1 1 | 7 1 1 | 8 1 | 5 3 1 | 1 1 5 2 | 6 1 1 1 | 2 3 2 2 |

infusion, whilst others reported pain elsewhere in the arm. There was no redness or swelling at the site of infusion or discolouration over the infusion vein and no evidence of extravasation. Slowing or discontinuation of the infusion usually led to rapid resolution of the pain; it was often possible to re-start DACA at a slower infusion rate and then increase the rate of infusion. At the 800 mg m$^{-2}$, however, the arm pain was more consistent and more severe.

Several patients also described a pattern of side-effects that were unusual but not severe. One patient treated at the 450 mg m$^{-2}$ dose level, reported feeling sleepy (grade 2); at the 600 mg m$^{-2}$ dose level another felt drowsy and on another occasion uncoordinated and 'disembodied' (grade 1). Paraesthesia (grade 1) around the mouth was seen in one patient at 450 mg m$^{-2}$ dose level. At the 600 mg m$^{-2}$ dose level, another patient reported burning around the mouth, face, eyes and throat associated with excessive lacrimation (grade 2). At the 800 mg m$^{-2}$ dose level, several patients described transient pain or paraesthesia around the mouth, altered taste, flushing or a sensation of anxiety.

A 36-year-old woman, treated at the 800 mg m$^{-2}$ dose level through a central venous catheter, experienced chest pain 30 min after the infusion started. This was associated with a burning sensation extending from her neck to her throat and mouth, and paraesthesia. Despite glyceryl trinitrite, after the infusion had been running for 190 min the central chest pain became severe (grade 3) with heaviness and tingling in her left arm. An ECG showed flattening of the T waves in the chest leads. The symptoms and ECG changes were suggestive of myocardial ischaemia. After the infusion was stopped and i.v. diamorphine given, the pain and ECG changes resolved rapidly; there were no changes in cardiac enzymes. She had no prior history of cardiovascular disease and did not experience further chest pain, but was not given further DACA.

Clinically significant myelosuppression was rare. There was no dose-limiting myelosuppression at dose levels up to and including 600 mg m$^{-2}$ with only one patient (treated at the 350 mg m$^{-2}$ dose level) developing grade 1 thrombocytopenia. At the 800 mg m$^{-2}$ dose level one patient had grade 1 neutropenia; a second patient experienced transient uncomplicated grade 4 thrombocytopenia and neutropenia on day 11 of cycle 2. Although this fulfilled the definition of DLT, it was transient and uncomplicated. It should also be noted that this patient had previously undergone high-dose chemotherapy with peripheral stem cell support and may have been more susceptible to myelosuppression. There were no episodes of grade 3 or grade 4 infection or fever related to DACA.

Although prophylactic antiemetics were not administered, nausea and vomiting was generally mild. One patient at the 800 mg m$^{-2}$ dose level had grade 3 vomiting that led to discontinuation of DACA but resolved following ondansetron. No significant changes in urea and electrolytes, calcium or liver biochemistry tests were observed in relation to DACA.

### Pharmacokinetics

Thirty six patients had samples collected after their first infusion of DACA and 34 had sampling on day 3. The mean number of samples taken on day 1 and day 3 were 17 and 18 respectively. Patients who did not receive the full dose of DACA were excluded from the pharmacokinetic analysis for that day.

Most patients' data fitted best a two-compartment model, but day 1 data from five patients fitted a one-compartment model better. A typical concentration-time curve is shown in Figure 2. There was a linear relationship between dose and AUC (Figure 3) on both day 1 ($r^2$ = 0.77, $P$ = 0.0005) and day 3 ($r^2$ = 0.64, $P$ = 0.006). There was also a linear relationship between dose and peak plasma concentration on both day 1 ($r^2$ = 0.57, $P$ = 0.007)

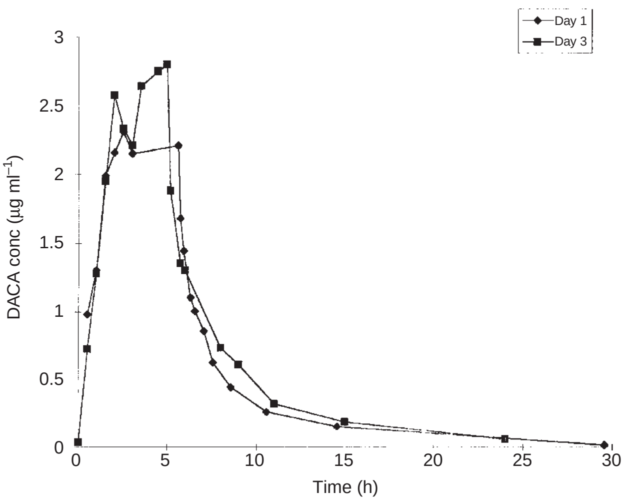

**Figure 2** Concentration-time curve for DACA

**Table 3** Summary of pharmacokinetic parameters for day 1

| Dose (mg m$^{-2}$) | AUC$_\infty$ (µg ml$^{-1}$ h) | Cl (l h$^{-1}$) | MRT (h) | $C_{max}$ (µg ml$^{-1}$) | $T_{max}$ (h) |
|---|---|---|---|---|---|
| 9 | 0.20 ± 0.03 | 72.79 ± 2.91 | 0.98 ± 0.16 | 0.08 ± 0.007 | 3.08 ± 0.11 |
| 18 | 0.48 ± 0.23 | 85.14 ± 54.8 | 1.82 ± 0.84 | 0.09 ± 0.018 | 2.66 ± 0.29 |
| 36 | 0.82 ± 0.39 | 99.24 ± 46.9 | 1.93 ± 1.13 | 0.24 ± 0.076 | 3.08 ± 0.14 |
| 60 | 1.62 ± 0.69 | 76.14 ± 30.6 | 1.23 ± 0.28 | 0.51 ± 0.229 | 3.06 ± 0.09 |
| 100 | 2.15 ± 0.46 | 92.35 ± 27.6 | 1.64 ± 0.63 | 0.56 ± 0.106 | 2.42 ± 0.69 |
| 165 | 3.52 ± 0.51 | 92.97 ± 37.1 | 1.05 ± 0.29 | 0.97 ± 0.176 | 2.79 ± 0.51 |
| 250 | 6.59 ± 1.04 | 64.60 ± 3.56 | 1.23 ± 0.03 | 2.22 ± 0.328 | 2.92 ± 0.39 |
| 350 | 7.72 ± 1.03 | 78.11 ± 10.9 | 1.64 ± 0.82 | 2.09 ± 0.115 | 3.17 ± 0.83 |
| 450 | 8.05 ± 2.12 | 98.85 ± 15.5 | 1.38 ± 0.23 | 2.38 ± 0.74 | 2.75 ± 0.58 |
| 600 | 16.3 ± 4.15 | 82.90 ± 24.9 | 2.78 ± 2.54 | 3.77 ± 0.48 | 2.33 ± 0.57 |
| 800 | 21.5 ± 10.7 | 76.26 ± 34.1 | 2.43 ± 1.33 | 4.84 ± 4.09 | 3.72 ± 1.67 |

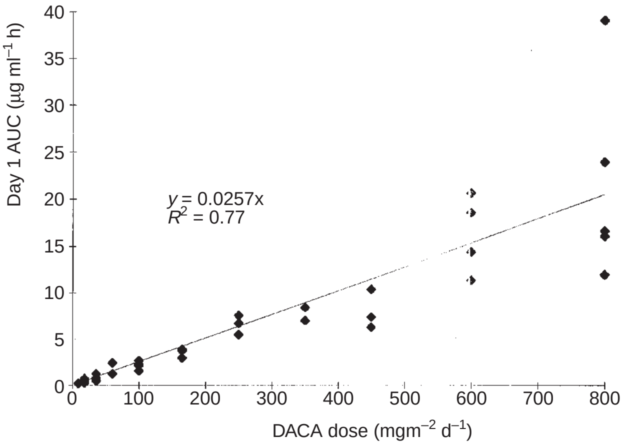

**Figure 3** Relationship between DACA dose and AUC

and day 3 ($r^2$ = 0.78, $P$ = 0.0001). There were no significant correlations between dose and either clearance or $V_{ss}$. Table 3 shows a summary of pharmacokinetic parameters for day 1. Across all dose levels there were no differences between day 1 and day 3 DACA plasma clearance (83.9 and 81.11 h$^{-1}$ respectively), MRT (1.74 and 1.91 h respectively), $T_{max}$ (2.91 and 2.69 h respectively), $V_{ss}$ (142.6 and 145.21 respectively) and $T_{1/2\beta}$ (2.22 and 2.08 h respectively).

The patient who experienced chest pain had a $C_{max}$ of 3.15 µg ml$^{-1}$, similar to that of other patients treated at the 800 mg m$^{-2}$ dose level (range 2.31–12.1 µg ml$^{-1}$). There was no significant relationship between either $C_{max}$ or AUC and severity of arm pain during infusion of DACA. Interestingly, the only patient with significant myelosuppression had the highest AUC on day 3 of all patients in the study (54.2 µg ml$^{-1}$ h).

### Anti-tumour activity

There were no objective responses to DACA according to WHO criteria. Seven patients treated at doses between 60 and 800 mg m$^{-2}$ had stable disease with progression-free intervals between 6 and 20 weeks. These included one patient with breast cancer and multiple small pulmonary metastases who received DACA 800 mg m$^{-2}$. Repeat computerized tomography scans showed a reduction in the number and size of these lesions, but this did not satisfy WHO criteria for a partial response.

### DISCUSSION

Many of the cytotoxic drugs in clinical use act against one or other of the DNA topoisomerases I and II. Preclinical data suggest that it may be worthwhile to combine inhibitors of topoisomerase I and II. Tan et al (1989) derived separate P388 leukaemia cell lines resistant to topoisomerase I or II. The topoisomerase I-resistant variants had reduced topoisomerase I levels but increased levels of topoisomerase II. Likewise, topoisomerase II levels were reduced in the topoisomerase II-resistant line but topoisomerase I levels were elevated. There was a similar pattern in Chinese hamster ovary cells (Gupta et al, 1988), A549 human lung cancer and HT29 colon cancer cell lines (Sugimoto et al, 1990). These data suggest that resistance to inhibitors of one topoisomerase may be associated with increased sensitivity to inhibitors of the other and raises the possibility of synergy between topoisomerase I and II inhibitors. This beneficial effect on cytotoxicity is seen in some human cell lines (Kano et al, 1992) but not in others (Kaufman et al, 1991).

In the clinic, attempts to combine topoisomerase I and II inhibitors have had limited success. Until recently, actinomycin D was the only single agent with demonstrable activity against topoisomerases I and II (Tewey et al, 1984; Trask and Muller, 1988). In a phase I trial of intoplicine, a 7-H-benzo[*e*]pyridol[4,3-*b*]-indole derivative that also has activity against topoisomerases I and II, hepatotoxicity was dose-limiting (Abigerges et al, 1996). When the established inhibitors of topoisomerase I and II irinotecan and etoposide were combined, diarrhoea and myelosuppression were severe (Karato et al, 1993). Although the combination was active, the toxicities were considered unacceptable despite granulocyte colony-stimulating factor support. As a novel inhibitor of topoisomerases I and II, DACA offers a different approach. DACA is active in several preclinical tumour models and a striking feature is the ability of DACA to overcome a number of mechanisms of drug resistance.

In this study, DACA was generally well-tolerated at all but the highest dose level of 800 mg m$^{-2}$ given on 3 successive days. The DLT was pain felt in the arm during infusion. In contrast to most cytotoxic agents, toxicities such as alopecia, stomatitis, myelosuppression and nausea and vomiting were uncommon. The arm pain occurred at all dose levels, but varied in intensity between patients at each dose level and also between infusions in the same patients. In all cases, the pain was reversible and there were no sequelae. At the highest dose level, flushing, perioral paraesthesia and pain were also seen.

 © 1999 Cancer Research Campaign

The pattern of toxicities seen with DACA is unusual and the mechanism unclear. The arm pain cannot be attributed to thrombophlebitis, as it was not limited to the course of the vein, and there were no visible changes in the skin overlying the vein. In no case was the pain associated with extravasation and in many patients the pain extended proximally up the arm. Attempts to abrogate this pain by the use of prophylactic analgesics and vasoactive substances were unsuccessful. The pain was often worst early in the infusion and settled when the infusion rate was slowed. This probably explains the absence of any relationship between $C_{max}$ and the arm pain. Nevertheless, the pain clearly was dose-related and determined by the mode of administration. One explanation for the arm pain would be vasoconstriction, but DACA appears not to be a potent vasoconstrictor (Xenova, personal communication). An alternative explanation would be action on local pain receptors, either directly or by stimulating release of peptide. This would be compatible with the 'tachyphylaxis' seen during the infusion and might also explain the perioral symptoms and flushing. Another possibility is an effect on plasma-ionized calcium levels.

Clearly in this study peripheral venous administration of DACA was not tolerable at the highest dose levels. Central venous administration would have been an alternative, but the only patient who received DACA by this route experienced chest pain associated with ECG changes. Although the pain was cardiac in nature, both the pain and ECG changes resolved rapidly following discontinuation of the infusion and there was no evidence of myocardial infarction. The mechanism of chest pain is again unclear. Nevertheless, the administration of DACA through a central venous catheter at this dose and using the current schedule would be inappropriate.

The difficulties in administering DACA as a peripheral infusion may explain the limited evidence of anti-proliferative activity. There were no objective tumour responses but a woman with breast cancer treated at the 800 mg $m^{-2}$ dose level had a reduction in the number and size of her pulmonary metastases. This did not, however, satisfy standard criteria for a partial response. This patient also experienced dose-limiting myelosuppression, but had previously undergone high-dose chemotherapy and was probably more susceptible to myelosuppression. Nevertheless, it appears that using this schedule, a dose of 800 mg $m^{-2}$ on 3 successive days may be close to that which has significant anti-proliferative activity.

In a second phase I trial of DACA conducted in New Zealand DACA was given as a 3-h infusion on a single day. Arm pain was again the DLT (B Evans, personal communication). These trials raise the question of how higher doses of DACA, which may have useful anti-tumour activity, can be administered. If the toxicities related to delivery can be overcome, it should be possible safely to escalate DACA doses further as the pharmacokinetic data have confirmed that DACA kinetics are linear over the dose range studied. With no evidence of time dependency in DACA pharmacokinetics between day 1 and day 3, it may be possible to administer DACA over a more prolonged period. This would reduce the peak levels of DACA to which patients are exposed.

In conclusion, we have demonstrated that the MTD for DACA given as a 3-hour i.v. infusion on 3 successive days repeated every 3 weeks is 800 mg $m^{-2}$ $day^{-1}$. The DLT was arm pain and the MTD was determined by symptoms related to mode of administration, which would have made further treatment unacceptable. Alternative means of delivery should be explored with DACA in future phase I studies.

### ACKNOWLEDGEMENTS

We thank the CRC Phase I/II committee, Data Centre and Dr David Secher (Director of Drug Development) for their support.

### REFERENCES

Abigerges D, Armand J-P, Chabot GG, Bruno R, Bissery M-C, Bayssas M, Klink-Alaki M, Clavel M and Catimel G (1996) Phase I and pharmacology study of intoplicine (RP 60475; NSC 645008), a novel topoisomerase I and II inhibitor, in cancer patients. *Anticancer Drugs* **7**: 166–174

Atwell GJ, Rewcastle GW, Baguley BC and Denny WA (1987) Potential anti-tumour agents. 50. In vivo solid tumour activity of derivatives of N-[2-(dimethylamino)ethyl]acridine-4-carboxamide. *J Med Chem* **30**: 664–669

Baguley BC, Holdaway KM and Fray LM (1990) Design of DNA intercalators to overcome topoisomerase II-mediated multi-drug resistance. *J Natl Cancer Inst* **82**: 398–402

Baguley BC, Zhuang L and Marshall E (1995) Experimental solid tumour activity of N-[2-(dimethylamino)ethyl]acridine-4-carboxamide. *Cancer Chemother Pharmacol* **36**: 224–248

Evans SMH, Young D, Robertson IGC and Paxton JW (1992) Intraperitoneal administration of the anti-tumour agent of N-[2-(dimethylamino) ethyl] acridine-4-carboxamide in the mouse: bioavailability, pharmacokinetics and toxicity after a single dose. *Cancer Chemother Pharmacol* **31**: 32–36

Finlay GJ and Baguley BC (1989) Selectivity of N-[2-(dimethylamino) ethyl] acridine-4-carboxamide towards Lewis lung carcinoma and human tumour cell lines in vitro. *Eur J Cancer Clin Oncol* **25**: 271–277

Finlay GJ, Marshall E, Matthews JHL, Paul KD and Baguley BC (1993) In vitro assessment of N-[2-(dimethylamino) ethyl] acridine-4-carboxamide, a DNA-intercalating antitumour drug with reduced sensitivity to multidrug resistance. *Cancer Chemother Pharmacol* **31**: 401–406

Gupta RS, Gupta R, Eng B, Lock RB, Ross WE, Hertzberg RP, Caranfa MJ and Johns RK (1988) Camptothecin-resistant mutants of Chinese hamster ovary cells containing a resistant form of topoisomerase I. *Cancer Res* **48**: 6404–6410

Kano Y, Suzuki K, Akatsu M, Suda K, Inoue Y, Yoshida M, Sakamoto S and Miura Y (1992) Effects of CPT-11 in combination with other anti-cancer agents in culture. *Int J Cancer* **50**: 604–610

Karato A, Saski Y, Shiraishi J et al (1993) Phase I study of CPT-11 and etoposide in patients with refractory solid tumours. *J Clin Oncol* **11**: 2030–2035

Kaufman SH (1991) Antagonism between camptothecin and topoisomerase II-directed chemotherapeutic agents in a human leukaemia cell line. *Cancer Res* **51**: 1129–1136

Marshall ES, Finlay GJ, Matthews JHL, Shaw JHF, Nixon J and Baguley BC (1992) Microculture-based chemosensitivity testing: a feasibility study comparing freshly explanted human melanoma cells with human melanoma cell lines. *J Natl Cancer Inst* **84**: 340–345

Paxton JW, Young D, Evans SMH, Kestell P, Robertson IGC and Cornford EM (1992) Pharmacokinetics and toxicity of the anti-tumour agent N-[2-(dimethylamino) ethyl]acridine-4-carboxamide after i.v. administration in the mouse. *Cancer Chemother Pharmacol* **29**: 379–384

Paxton JW, Young D, Evans SMH, Robertson IGC and Kestell P (1993*a*) Tumour profile of N-[2-(dimethylamino) ethyl]acridine-4-carboxamide after intraperitoneal administration in the mouse. *Cancer Chemother Pharmacol* **32**: 320–322

Paxton JW, Young D and Robertson IGC (1993*b*) Pharmacokinetics of acridine-4-carboxamide in the rat, with extrapolation to humans. *Cancer Chemother Pharmacol* **32**: 323–325

Schneider E, Darkin SJ, Lawson PA, Ching L.-M, Ralph RK and Baguley BC (1988) Cell line selectivity and DNA breakage properties of the anti-tumour agent N-[2-(dimethylamino) ethyl]acridine-4-carboxamide: role of DNA topoisomerase II. *Eur J Cancer Clin Oncol* **24**: 1783–1790

Tewey KM, Chen GL, Nelson EM and Liu LF (1984) Intercalative antitumour drugs interfere with the breakage-reunion reaction of mammalian DNA topoisomerase II. *J Biol Chem* **259**: 9182–9187

Trask DK and Muller MT (1988) Stabalization of type I topoisomerase-DNA covalent complexes by actinomycin D. *Proc Natl Acad Sci USA* **85**: 1417–1421

© 1999 Cancer Research Campaign 
